# MicroRNA as Epigenetic Modifiers in Endometrial Cancer: A Systematic Review

**DOI:** 10.3390/cancers13051137

**Published:** 2021-03-06

**Authors:** Amélia Favier, Grégoire Rocher, Annette K. Larsen, Romain Delangle, Catherine Uzan, Michèle Sabbah, Mathieu Castela, Alex Duval, Céline Mehats, Geoffroy Canlorbe

**Affiliations:** 1 Centre de Recherche Saint-Antoine (CRSA), INSERM UMR_S_938, Cancer Biology and Therapeutics, Sorbonne University, 75012 Paris, France; annette.larsen@mfex.com (A.K.L.); catherine.uzan@aphp.fr (C.U.); michele.sabbah@inserm.fr (M.S.); 2 Department of Gynecological and Breast Surgery and Oncology, Pitié-Salpêtrière, Assistance Publique des Hôpitaux de Paris (AP-HP), University Hospital, 75013 Paris, France; gregoire.rocher@gmail.com (G.R.); romaindelangle@hotmail.fr (R.D.); 3 Centre de Recherche Saint-Antoine, Equipe Instabilité des Microsatellites et Cancer, Equipe labellisée par la Ligue Nationale contre le Cancer, Unité Mixte de Recherche Scientifique 938 and SIRIC CURAMUS, INSERM, Sorbonne Université, 75012 Paris, France; alex.duval@inserm.fr; 4 Scarcell Therapeutics, 101 rue de Sèvres, 75006 Paris, France; castela.mathieu@yahoo.fr; 5 U1016, CNRS, UMR8104, Institut Cochin, INSERM, Université de Paris, 75014 Paris, France; celine.mehats@inserm.fr

**Keywords:** Epigenetics, MicroRNA, Endometrial cancer, Methylation, miR-182, miR-230, miR-129-2, miR-130a/b, miR-200b, miR-191

## Abstract

**Simple Summary:**

Endometrial cancer (EC) is the 2nd most common gynecologic cancer worldwide. MicroRNAs (miRNAs) are small noncoding RNAs that contribute to epigenetic regulation. The objective of this systematic review is to summarize our current knowledge on the role of miRNAs in the epigenetic deregulation of tumor-related genes in EC. It includes all miRNAs reported to be involved in EC including their roles in DNA methylation and RNA-associated silencing. This systematic review should be useful for development of novel strategies to improve diagnosis and risk assessment as well as for new treatments aimed at miRNAs, their target genes or DNA methylation.

**Abstract:**

The objective of this systematic review is to summarize our current knowledge on the influence of miRNAs in the epigenetic deregulation of tumor-related genes in endometrial cancer (EC). We conducted a literature search on the role of miRNAs in the epigenetic regulation of EC applying the Preferred Reporting Items for Systematic Reviews and Meta-Analyses (PRISMA) guidelines. The following terms were used: microRNA, miRNA, miR, endometrial cancer, endometrium, epigenetic, epimutation, hypermethylation, lynch, deacetylase, DICER, novel biomarker, histone, chromatin. The miRNAs were classified and are presented according to their function (tumor suppressor or onco-miRNA), their targets (when known), their expression levels in EC tissue vs the normal surrounding tissue, and the degree of DNA methylation in miRNA loci and CpG sites. Data were collected from 201 articles, including 190 original articles, published between November 1, 2008 and September 30, 2020 identifying 313 different miRNAs implicated in epigenetic regulation of EC. Overall, we identified a total of 148 miRNAs with decreased expression in EC, 140 miRNAs with increased expression in EC, and 22 miRNAs with discordant expression levels. The literature implicated different epigenetic phenomena including altered miRNA expression levels (miR-182, -230), changes in the methylation of miRNA loci (miR-34b, -129-2, -130a/b, -152, -200b, -625) and increased/decreased methylation of target genes (miR-30d,-191). This work provides an overview of all miRNAs reported to be involved in epigenetic regulation in EC including DNA methylation and RNA-associated silencing. These findings may contribute to novel strategies in diagnosis, risk assessment, and treatments aimed at miRNAs, their target genes or DNA methylation.

## 1. Introduction

With 417,367 new cases and 97,370 deaths each year, endometrial cancer (EC) is the 2nd most common gynecologic cancer worldwide after breast cancer [1].

Although the mechanisms underlying endometrial carcinogenesis are not fully understood, current evidence suggests that alterations of the epigenome drive both the expression of oncogenes and downregulation of tumor suppressors thereby promoting tumor initiation and progression in EC. Three epigenetic systems are currently known to modify gene expression: DNA methylation, histone modifications and RNA-associated silencing [2,3,4,5,6].

Micro-RNAs (miRNAs) are a family of small noncoding RNAs, 21–25 nucleotides in length that are involved in epigenetic mechanisms. miRNAs are transcribed by RNA polymerase II [7] or III [8] as long primary transcripts (pri-miRNAs) in the nucleus and then cleaved by RNAse III to become miRNAs. Each miRNA has the potential to regulate a variety of genes (usually around 500), while each gene is typically targeted by several different miRNAs [9,10,11,12]. It is well recognized that miRNAs are important regulators of genes which play crucial roles in fundamental biological processes such as proliferation, differentiation and survival [13,14,15,16,17]. miRNAs may also play a decisive role in the control of chromatin structure and gene expression by directly influencing the post-transcriptional regulation of important chromatin- and DNA-modifying enzymes. Recent studies have shown that a number of miRNAs, including miR-129-2 and miR-152, can be epigenetically silenced by hyper-methylation of their respective DNA locus in EC [14,18,19,20,21,22,23,24,25]. It has also been reported that miR-191 is able to down- or upregulate the level of methylation of certain genes in EC [14].

We recently published a systematic review focusing on the use of miRNAs in the management of EC [26]. In contrast, there is, to the best of our knowledge, no recent review of the role of miRNA as epigenetic modifiers in EC. In this review, we cover all epigenetic phenomena that have been correlated with a role for miRNAs in the tumorigenesis of EC [6,26] including the influence of miRNAs on the expression of tumor-related genes.

## 2. Methods

**Figure 1:**
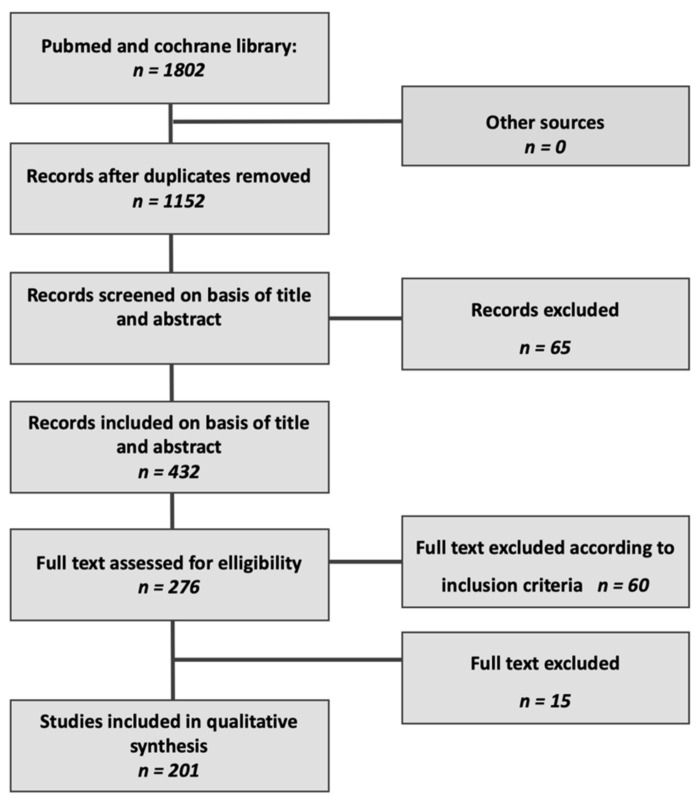
PRISMA flow diagram.

This systematic review was carried out using the following databases following the Preferred Reporting Items for Systematic Reviews and Meta-Analyses (PRISMA) guidelines (Figure 1):

MEDLINE, PubMed (the Internet portal of the National Library of Medicine, http://www.ncbi.nlm.nih.gov/sites/entrez?db=pubmed; accessed on 30 September 2020), the Cochrane Library, Cochrane databases “Cochrane Reviews”, and “Clinical Trials” (http://www3.interscience.wiley.com/cgi-bin/mrwhome/106568753/HOMEDARE; accessed on 30 September 2020). 

We used the following terms: microRNA, miRNA, miR, endometrial cancer, endometrium, epigenetic, epimutation, hypermethylation, lynch, deacetylase, DICER, novel biomarker, histone, chromatin.

The database search was further supplemented with original articles, reviews, and meta-analyses, including the studies cited therein. Only articles published in English or French between November 1, 2008 and September 30, 2020 were included.

The miRNAs are presented according to their expression levels in EC tissue compared to the healthy surrounding tissues, their function (tumor suppressor or oncomiRNA), and the degree of DNA methylation in miRNA loci and in CpG islands of target genes.

## 3. Results

Data were collected from 190 original articles and 11 reviews identifying a potential role for 313 different miRNAs in EC.

**Figure 2:**
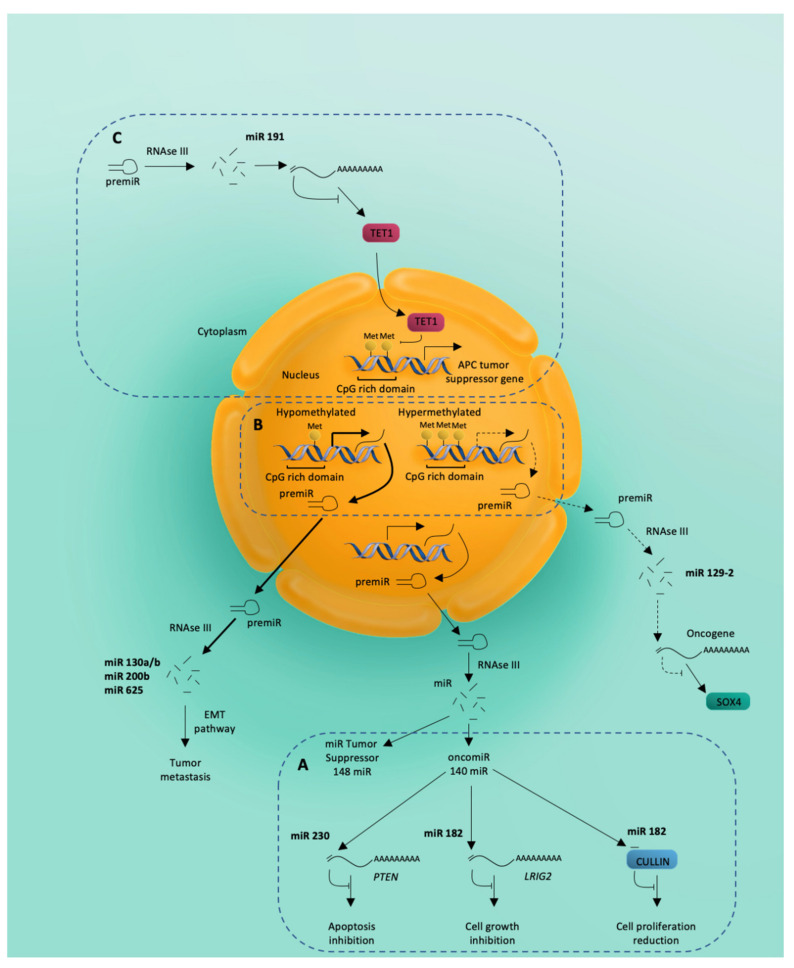
MicroRNA (miRNA) as epigenetic modifiers in endometrial cancer. Schematic summary of the three epigenetic mechanisms known to involve miRNAs. (**A**) miRNAs can act as tumor suppressors or as oncomiRNAs. miR-200 binds directly to PTEN resulting in the inhibition of apoptosis. miR-182 binds directly to LRIG2 leading to inhibition of cell growth while the miR-182-mediated decrease in cullin-5 protein levels results in increased cell proliferation. (**B**) CpG-rich domains of miRNA loci can be hypo- or hyper-methylated. Hyper-methylation of the miR-129-2 locus leads to decreased expression of this miRNA, which acts as a natural inhibitor of the SOX4 oncogene, thereby activating SOX4. (**C**) TET1 expression is downregulated by miR-191 through the mRNA-miRNA interaction in the 3’-untranslated region of TET1. Downregulation of the TET1 protein is accompanied by hyper-methylation of the promotor region of tumor suppressors like adenomatous polyposis coli (APC), resulting in decreased gene expression and attenuated levels of APC protein.

A model of the different roles of miRNA as epigenetic modifiers in EC is shown in Figure 2 with the three epigenetic mechanisms known to involve miRNAs. Figure 2A miRNAs can act as tumor suppressors or as oncomiRNAs. Figure 2B CpG-rich domains of miRNA loci can be hypo- or hyper-methylated. Figure 2C TET1 expression is downregulated by miR-191 through the mRNA–miRNA interaction in the 3′-untranslated regions of TET1.

### 3.1. Expression Profile of miRNAs of Epigenetic Modifiers Associated with Malignant Endometrial Tissue Compared with Healthy Endometrial Tissue

A summary of these data is provided in Table 1.

This systematic review identified 105 original articles and two literature reviews reporting the expression pattern of miRNAs [27,28,29,30,31,32,33,34,35,36,37,38,39,40,41,42,43,44,45,46,47,48,49,50,51,52,53,54,55,56,57,58,59,60,61,62,63,64,65,66,67,68,69,70,71,72,73,74,75,76,77,78,79,80,81,82,83,84,85,86,87,88,89,99,100,101,102,103,104,105,106,107,108,109,110,111,112,113,114,115,116,117,118,119,120,121,122,123,124,125,126,127,128,129,130,131,132,133,134,135]. Of the 69 articles comparing neoplastic endometrial tissue with the surrounding healthy tissue, 39 were published after October 31, 2018 and were not presented in our previous review [27,28,29,30,31,32,33,34,35,36,37,38,39,40,41,42,43,44,45,46,47,48,49,50,53,54,55,56,57,58,59,61,62,63,64,65,66,67,68,69,70,71,72,73,74,75,76,77,78,79,80,81,82,83,84,85,86,87,88,94,96,98,99,100,101,102,103,131,132,133,134]. Overall, the 69 studies included an average of 82 endometrial tumor samples (minimum 4, maximum 579) and 22 healthy endometrial samples (minimum 5, maximum 56). Nineteen articles reported that the miRNAs were extracted from paraffin and 26 from frozen tissue. On-chip hybridization techniques (15 articles) or direct RT-qPCR (96 articles) were used to measure the expression levels of the miRNAs. We identified 148 miRNAs with decreased expression in EC, 140 miRNAs with increased expression in EC, and 22 miRNAs with discordant expression levels (Table A1).

Endometrial tumors showed the following miRNA expression levels compared with healthy endometrial tissues:

-Increased miRNA expression: miR-7, miR-let-7a, miR-let-7f, miR-let-7g, miR-9, miR-9-3p, miR-10a, miR-17, miR-18a-3p, miR-19b, miR-25-5p, miR-27a, miR-30d, miR-31, miR-34a, miR-95,miR-96, miR-103, miR-106a, miR-106b, miR-106b-93-25, miR-107, miR-129-2, miR-130b, miR-135a, miR-135b, miR-141, miR-142-5p, miR-145, miR-146, miR-146b-5p, miR-150, miR-151, miR-153, miR-155, miR-181a, miR-181c-3p, miR-181c, miR-182, miR-183, miR-183-3p, miR-184, miR-185a, miR-185, miR-186, miR-191, miR-193-3p, miR-194, miR-200 family (miR-200a, miR-200b…), miR-203, miR-205, miR-210, miR-215, miR-219-2, miR-221, miR-223, miR-218, miR-301, miR-325, miR-326, miR-330, miR-331, miR-331-3p, miR-337, miR-363, miR-373, miR-423, miR-425, miR-429, miR-432, miR-449, miR-449a, miR-499, miR-518d-5p, miR-520c-5p, miR-522, miR-526a, miR-616, miR-625, miR-874, miR-891a, miR-940, miR-1202, miR-1224, miR-1269, miR-5787 and miR-6749-5p

-Decreased miRNA expression: miR-Let-7c, miR-1-2, miR-6, miR-10b, miR-15b, miR-20a-5p, miR-20b-5p, miR-21, miR-21-5p, miR-23a*, miR-27b-3p, miR-29c, miR-29c-3p, miR-29b, miR-30a-3p, miR-30a-5p, miR-30c, miR-31, miR-32, miR-33b, miR-34b, miR-99a, miR-99a-3p, miR-99b, miR-100, miR-101, miR-101-2, miR-107-5p, miR124, miR-126, miR-127-3p, miR-130b, miR-133, miR-133b, miR-136, miR-137, miR-139, miR-139-5p, miR-142, miR-143, miR-145, miR-144-3p, miR-146a, miR-148b, miR-149, miR-152, miR-184, miR-185, miR-185-5p, miR-193b, miR-193, miR-193a-5p-YY1-APC, miR-194, miR-195, miR-196a, miR-196a-5p, miR-197, miR-199b, miR-199b-3p, miR-199b-5p, miR-202-3p, miR-203, miR-204, miR-204-5p, miR-205-5p, miR-214, miR-214-3p, miR-216b, miR-221, miR-302a-5p, miR-328-3p, miR-320a, miR-335, miR-337-3p, miR-338-3p, miR-340-5p, miR-361, miR-367-3p, miR-368, miR-369, miR-370, miR-376a, miR-376c, miR-377, miR-377-5p, miR-381, miR-409, miR-410, miR-411, miR-424, miR-424*, miR-424-3p, miR-431, miR-432, miR-449a, miR-451, miR-455-5p, miR-483-5p, 487b, miR-495, miR-496, miR-503, miR-516, miR-516b, miR-542-3p, miR-542-5p, miR-543, miR-589-5p, miR-596, miR-610, miR-630, miR-632, miR-638, miR-646, miR-652, miR-758, miR-760, miR-874, miR-1247, miR-1296, miR-3926-1, miR-4429, miR-4461, miR-6076 and miR-6511b

### 3.2. DNA Methylation Levels of miRNA Loci in Malignant and Healthy Endometrial Tissues

A summary of these data is presented in Table 2.

We identified nine articles [21,22,23,24,25,135,136,137,138] which studied the degree of DNA methylation in miRNA loci. Among these, eight articles compared neoplastic endometrial tissue with the surrounding healthy tissue [22,23,24,25,135,136,137,138], while one article compared cancer tissues from various stages [21].

The following techniques were used to determine the relative methylation levels of miRNA loci: combined bisulfite restriction analysis (COBRA) using the DNA methylation kit (Zymo^®^, Research, Irvine, CA, USA), the EpiTect Bisulfite Kit (Qiagen^®^, Valencia, CA, USA) (a methylation sodium bisulfite kit), anti-5-methylcytosine monoclonal antibodies, methylation-specific multiplex ligation-dependent probe amplification, 5-aza-2′-deoxycytidine (5-AZA; a DNA methylation inhibitor) and/or Trichostatin A (TSA; a histone deacetylase inhibitor).

Endometrial tumors showed the following degrees of methylation in miRNA loci compared with healthy endometrial tissue:

-miRNAs with hypo-methylated loci: miR-130a/b, miR-182, miR-200b, miR-208a, miR-222, miR-625

-miRNAs with hyper-methylated loci: miR-34b, miR-124a-1, miR-124a-2, miR-124a-3, miR-129-2, miR-137, miR-152, miR-638, miR-663

### 3.3. DNA Methylation Levels of miRNA Loci in Malignant Endometrial Iissue

A summary of these data is provided in Table 2.

We identified two articles that reported the DNA methylation levels of miRNA target genes [14,139].

Two techniques were used to determine the relative methylation level of the miRNA target genes: immunoprecipitation of hydroxymethylated DNA followed by quantitative PCR, and anti-5-methylcytosine monoclonal antibodies.

Endometrial tumors showed the following alterations in the methylation of miRNA target genes:

-miR-30d increased the methylation of the H19 locus (although this miRNA does not act as a methyltransferase).

-miRNA-191 downregulated TET1 expression, an enzyme that is involved in the removal of methylated DNA in the loci of adenomatous polyposis coli (APC) and other tumor suppressor genes.

## 4. Discussion

This is the first systematic literature review on miRNAs in EC that focus on their roles in the control of chromatin structure and gene expression. We identified 148 miRNAs with decreased expression in EC, 140 miRNAs with increased expression in EC, and 22 miRNAs with discordant expression levels. In addition, endometrial tumors displayed six hypo-methylated and nine hyper-methylated miRNA loci in comparison to normal endometrial tissue. Finally, two miRNAs were reported to be involved in specific epigenetic phenomena: miR-30d was found to directly methylate the CpG promoter of the H19 gene while miR-191 was able to downregulate the expression of TET1, an enzyme that usually removes methylated bases in the promoter region of tumor suppressors like APC, thereby decreasing their expression [139].

In our previous review [26], we included 30 articles published between November 1, 2008 and October 31, 2018 studying the expression pattern of miRNAs in neoplastic endometrial tissue compared to healthy adjacent tissue. We described 110 miRNAs with decreased expression in EC, 133 with increased expression in EC, and 18 with discordant functions. The current systematic review includes 115 new original articles and three literature reviews published after November 1, 2018 emphasizing the strong current interest in this area. We here confirm the involvement of 313 miRNAs: (48 miRNAs with decreased expression, 140 with increased expression and 22 with discordant expression) including the four miRNAs most frequently involved in EC, miR-182, miR-183, the miR-200 family and miR-205 [28,46,68].

We previously described a role for miR-182 in the inhibition of cullin-5 which is accompanied by increased proliferation [110]. These findings were recently confirmed by Devor et al. who showed that miR-182 is often overexpressed in endometrial adenocarcinoma where it directly targets and inhibits cullin-5 [25]. Similarly, Jia et al. reported that LRIG2, a tumor suppressor gene mainly expressed in the ovaries and uterus, contain a putative binding site for miR-182 [140]. As a result, overexpression of miR-182 in EC overrides the inhibitory effects of LRIG2 on cell growth and the glycolytic metabolism [47].

We also reported that overexpression of miR-183 is associated with a poorer prognosis for EC patients both in terms of overall survival and progression-free survival. These findings were confirmed by a recent study based on the cancer genome atlas for miRNA expression [99].

The miR-200 family is implicated in the PI3K/AKT/mTOR signaling pathway, at least in part through downregulation of the PTEN tumor suppressor [141,142,143]. This was recently confirmed by Chen et al. who reported that miR-200c binds directly to PTEN and PTENP1 in endometrioid EC [102]. In agreement, a different study reported that estrogen stimulation increases the expression of miR-200c which is accompanied by decreased PTEN expression and activation of the PI3K-AKT pathway thereby promoting increased cellular survival [67].

In our previous study, we highlighted a potential prognostic role for miR-205. Since then, Zhao et al. have reported that miR-205 is closely related to overall survival using the Cancer Genome Atlas database that includes 164 miRNAs implicated in EC [42]. Donkers et al. also observed that miR-205 is consistently upregulated in EC. However, since miR-205 is also upregulated in lung and ovarian cancer [144,145], miR-205 might not be useful by itself as a diagnostic test for EC, although it may still serve as a prognostic biomarker.

Various studies have focused on the involvement of miRNAs in EC [26,146] but without exploring the underlying epigenetic mechanisms. miRNA levels can be increased or decreased in EC which, at least in part, can be due to differential methylation. To date, the methylation level of miRNA loci or of their target genes have been reported for only 17 miRNAs. However, differential methylation status has been reported for multiple genes in EC [147], indicating that epigenetic inactivation of gene promoters may be common in this disease. Importantly, aberrant DNA methylation appears to be more frequent in EC than genetic alterations. Interestingly, other studies have highlighted an association between hyper-methylation of six different miRNA loci (124a-1, 124a-3, 1-1, 148a, 152 and 18b) in other cancer types including gastric cancer as well as in colorectal cancers with microsatellite instability (MSI) [148,149,150].

Huang et al. reported that miR-129-2 functions as a tumor suppressor through negative regulation of SOX4, an oncogene frequently overexpressed in EC [138]. Importantly, methylation of the miR-129-2 locus was found in 68% of 117 EC patients with elevated SOX4 expression. Methylation of miR-129-2 has also been related to MSI and hypermethylated hMLH1. Therefore, oncogene activation may be caused either by methylation-mediated silencing of miRNA loci with an inhibitory action on oncogene expression and by direct demethylation of the oncogene promoter [142]. A better understanding of the methylation patterns of MMR genes that can be inherited over generations and may cause familial tumorigenesis such as Lynch syndrome (25% of MSI tumors) may lead to better treatment for these women who experience a 60% lifetime risk of EC [151].

A study by Tsuruta et al. highlighted a role for miR-152. Specifically, the expression of miR-152, that plays a role as a tumor suppressor can be reduced by aberrant DNA methylation. Treatment with 5-azacytidine, a demethylation agent, is able to restore the expression of miR-152. Aberrant methylation of the promoter of miR-152 has also been reported for other cancers including acute lymphoblastic leukemia, gastrointestinal cancer and cholangiocarcinoma [152,153,154,155].

We previously demonstrated that miR-137 is hyper-methylated in human endometrial tumors and confirmed that it acts as a tumor suppressor through epigenetic silencing [23]. Hyper-methylation of miR-137 was found in both endometrioid and serous endometrial cancer (*p* < 0.01), and was associated with loss of miR-137 expression. Hyper-methylation of the loci coding for miR-137 has also been reported for other cancers such as squamous cell carcinoma of the neck and head [156,157] and is associated with poorer overall survival [158]. The presence of MiR-137 in bowel lavage fluid is used as a prognostic marker for colorectal cancer, in oral rinses for head and neck squamous cell carcinoma, and in urine for bladder cancer [159,160].

miR-130a/b, miR-200b and miR-625 contain several CpG sites in their loci. The miR-130b and miR-200 family are involved in the regulation of the epithelial-mesenchymal transition pathway and tumor metastasis. Li et al. assessed the methylation status of these CpG islands in both endometrioid EC and normal endometrial tissue and reported that they were hypo-methylated in EC. The expression of miR-130b increased in EC cells after treatment with demethylation agents [135].

Moreno-Moya et al. showed another epigenetic phenomenon used by miRNAs: miR-30d is overexpressed in EC where it is able to methylate the H19 locus, which is associated with reproductive and endocrine system disorders as well as epithelial cell proliferation. When the methylation is reversed, H19 is upregulated in endometrial epithelial cells [139]. Yang et al. also reported a role for miRNA in DNA methylation. They demonstrated that miR-191 was upregulated in EC tissue in comparison with the adjacent normal tissue and that its knockdown repressed EC cell proliferation. miR-191 targets TET1, a methylcytosine dioxygenase which functions in the removal of genome-wide methylation DNA marks, thereby decreasing the expression of TET1 in EC. This results in hypermethylation of the promoter region of APC, a tumor suppressor and other tumor suppressors, thereby decreasing their expression [14].

Over the last 5 years, up to 754 miRNAs [17] have been identified as potential biomarkers in EC, some of which are correlated with lymph node involvement, advanced FIGO stage, metastatic status, or histologic type [17,40,103,118,161,162]. Increasing our knowledge of the miRNA expression status or the methylation state of key miRNA loci may help to better stratify patients. It is important to note that the molecular classification of EC has revealed considerable heterogeneity of tumors with comparable histologic type and grade but with different genes and epimutations. Future research should also be directed toward matching miRNAs to molecular classification subgroups of tumors. For the molecular classification of EC, type I tumors include PTEN, beta-catenin and KRAS gene mutations. PTEN mutations represent 94% of the tumors in the subgroup “POLE” and 88% of the “MSI-hypermutated” tumor subgroup [163]. They are frequently detected in patients with aberrant methylation of the MLH1 promoter regions that cause inactivation of the mismatch repair (MMR) gene [164,165]. The miR-200 family, miR-183 and miR-21 have been shown to target the PTEN gene, and their expression levels in endometrial tissue can be used to predict the risk of tumor progression from endometrial hyperplasia to invasive EC [102,122]. TP53 mutations, corresponding to the “Copy Number High” subgroups, are more common in grade 3 tumors, which are associated with poorer survival than other tumor groups. miR-34a has been found to be downregulated in p53-mutated ovarian cancer; miR-34a being the direct target of the tumor suppressor p53 gene [166].

The current European pathologic classification of EC is probably not sufficiently accurate to predict recurrence risk, often leading to over- or under-treatment [167]. Sensitive and specific molecular prognostic biomarkers are needed to better adapt surgery and adjuvant therapies. In this setting, various studies have demonstrated that miRNA can be used as a diagnostic tool for nodal status or for lymphovascular space involvement [10,162]. Promising trials are underway to investigate the usefulness of these miRNAs, particularly in blood or urine samples, to improve the management of patients with EC. The GYNO-MIR (NCT03776630) clinical trial explores the potential of novel biomarkers based on plasma miRNAs for a better management of pelvic gynecologic tumors. The aim of this clinical trial is to validate a 5 miRNA index as a diagnostic marker to assess the risk of lymph node metastases in EC and ovarian cancer from patient plasma samples taken during surgery and one month later. The Urinary miRNA (NCT03824613) clinical trial explores the accuracy of the predictive value of miRNA in distinguishing EC patients from healthy subjects, and if miRNA correlates with the final histology and/or the subtype of EC. Nevertheless, while stage I and stage II EC are largely curable, more advanced malignancies often progress to a chemo- and radio-resistant phenotypes. Aberrant DNA methylation is widespread in endometrial tumors and is associated with chemoresistance. Although single-agent epigenetic therapies have demonstrated some efficacy, the combination of an epigenetic therapy with conventional chemotherapy holds a greater promise by restoring the sensitivity to chemotherapy in patients with chemo-resistant EC [168].

This systematic review has some specific limitations. First, our literature search yielded only a few studies covering the degree of DNA methylation. This might be explained by the fact that the involvement of miRNA involvement in DNA methylation is a relatively new field of research that is yet to be explored. The second limitation is the general lack of research articles focusing on *de novo* carcinogenesis of type II EC and the use of molecular classifiers of EC.

## 5. Conclusions

In this review, we provide an overview of all miRNAs reported to be involved in epigenetic regulation of EC. Further clarification with respect to which miRNA families are promoting oncogenesis, which miRNAs play a role as tumor suppressors and which miRNAs are directly involved in modification of DNA methylation constitute an exciting new area of research. Improved diagnosis, risk assessment, and treatment strategies based on miRNA represents a promising area but will require future research.

## Figures and Tables

**Table 1:** Differences in the expression profiles of microRNAs between malignant and healthy endometrial tissues.

Reference	Date	Review	Type of Review Article	miRNA Increased	miRNA Decreased	Case Sample	Case Control	Detection Technique
Wang et al. [27]	2020	J Obstet Gynecol Reprod Biol X	Original Article	miR-135a		Human cell lines	Scrambled negative control RNAs	RT-PCR
Wu et al. [28]	2020	J Comput Biol	Original Article		miR-449a,-145-5p	Endometrial cancer tissue (*n* = 77)	Adjacent healthy endometrial tissue (*n* = 22)	bioinformatics
Donkers et al. [29]	2020	Onco target	Literature Review	miR-205, -200c,-223,-182,-183,-200a,-135b,-429,-141,-200b,-200a*,-222,-141-3p,-200c-3p,-186,-200b*,-15b,-106a,-135a,-205-5p,-182-5p,-200b-3p,-92a,-9-5p,-27a,-210,-96,-194, -95,-155,-18a* miR-222-3p miR-96-5p miR-103 miR-151 miR-34a miR-92a-1* miR-887-5p miR-20a* miR-106b* miR-449a miR-17* miR-185 miR-1228 miR-146 miR-425 miR-1290 miR-205 miR-200c miR-223 miR-182 miR-183 miR-200a miR-135b miR-429 miR-141 miR-200b miR-200a* miR-222 miR-141-3p miR-200c-3p miR-186 miR-200b* miR-15b miR-106a miR-135a miR-205-5p miR-182-5p miR-200b-3p miR-92a miR-9-5p miR-27a miR-210 miR-96 miR-194 miR-95 miR-155 miR-18a* miR-222-3p miR-96-5p miR-103 miR-151 miR-34a miR-92a-1* miR-887-5p miR-20a* miR-106b* miR-449a miR-17* miR-185 miR-1228 miR-146 miR-425 miR-1290	miR-137,-129-3p,-410,-503,-1247,-376c,-377,-26a-5p,-214,-150-5p,-370, let-7f-5p, -26b-5p, let-7c-5p,-23b-3p,-125b-5p, -126-3p, -195-5p, -424-5p, -374a-5p, -let-7a-5p, -let-7e-5p, -125a-5p, -542-5p miR-337-5p miR-1305 miR-758 miR-300 miR-93 miR-125 miR-34 miR-30a-3p miR-301b miR-10b miR-195 miR-30a-5p miR-101 miR-10b* miR-133a miR-133b miR-152 miR-29b miR-34b miR-411	Endometrial cancer tissue		RT-PCR
Wang et al. [30]	2020	Radiol Oncol	Original Article		miR-144-3p	Endometrial cancer Cell lines		RT-PCR
Liu Y et al. [31]	2020	J Cell Mol Med.	Original Article		miR-646	Endometrial cancer tissue (*n* = 32)	Normal endometrial tissue (*n* = 26)	RT-PCR/bioinformatics
Zong et al. [32]	2020	J Cell Mol Med	Original Article		miR-136	Endometrial cancer tissue (*n* = 69)		RT-PCR
Zhang et al. [33]	2020	Oncol rep	Original Article		miR-320a, miR-340-5p	Endometrial cancer tissue (*n* = 8)	Adjacent healthy endometrial tissue	RT-PCR
Shi et al. [34]	2020	Biochem	Original Article		miR-6076	Endometrial cancer Cell lines		RT-PCR
Zhang et al. [35]	2020	Exp Cell Res	Original Article		miR-320a	Endometrial cancer tissue (*n* = 41)	Normal endometrial tissue (*n* = 7)	bioinformatics
Xin et al. [36]	2020	Am J Transl Res	Original Article		miR-205-5p	Endometrial cancer tissue (*n* = 42)	Normal endometrial tissue (*n* = 29)	RT-PCR
Wang et al. [37]	2020	Oncol Rep	Original Article	miR-21-5p		Endometrial cancer tissue (*n* = 160)	Adjacent healthy endometrial tissue	RT-PCR
Sato et al. [38]	2020	Tohoku J Exp Med	Original Article		miR Let-7c	Paclitaxel-resistant cell lines		RT-PCR
Dou et al. [39]	2020	Kaohsiung J Med Sci	Original Article	miR-335		Endometrial cancer tissue (*n* = 47)	Adjacent healthy endometrial tissue	RT-PCR
Wilczynski et al. [40]	2020	Acta Obstet Gynecol Scand	Original Article		miRNA-204, miRNA-424	Endometrioid cancer tissue with lymphadenectomy positive	Endometrioid cancer tissue with lymphadenectomy negative	RT-PCR
Xu et al. [41]	2020	Cancer Biother Radiopharm	Original Article		miR-202-3p	Endometrial cancer type 2 (*n* = 20)		RT-PCR
Zhao et al. [42]	2020	J Cell Biochem	Original Article	miR-31,−205, −211,−425		Endometrial cancer tissue (*n* = 546)	Normal endometrial tissue (*n* = 33)	bioinformatics TCGA
Wang et al. [43]	2020	DNA Cell Biol	Original Article	hsa-miR-184,-4461	hsa-miR-6511b	Endometrial cancer tissue with recurrence (*n* = 232)	Endometrial cancer tissue with no recurrence (*n* = 231)	RT-PCR
Yang et al. [44]	2020	Cell Cycle	Original Article		miR-516b	Endometrial cancer tissue (*n* = 106)		RT-PCR
Wang et al. [45]	2020	J Cell Mol Med	Original Article	hsa-miR-183-3p, -200b-3p, -429, -1307-3p,-183-5p	miR-542-3p,-152-3p,-24-1-5p,-374b-5p	Endometrial cancer tissue (*n* = 441)	Normal endometrial tissue	bioinformatics (TCGA)
Liu et al. [46]	2020	Gene	Original Article		miR-149-5p	Endometrial cancer Cell lines		RT-PCR
Jia et al. [47]	2020	Cancer Cell International	Original Article	miR-182		Endometrial cancer tissue (*n* = 50)	Normal endometrial tissue (*n* = 30)	RT-PCR
Li et al. [48]	2019	Oncol Lett	Original Article		miR-23a	Endometrial cancer tissue (*n* = 16)	Adjacent healthy endometrial tissue	RT-PCR/immunohistochimie
Huang et al. [49]	2019	IUBMB	Original Article	miR-146b-5p		Endometrial cancer tissue with progesterone (*n* = 3) and without progesterone (*n* = 3)		RT PCR/bio informatics
Van Sinderen et al. [50]	2019	oncol Lett	Original Article		miR-29c	Endometrioid endometrial cancers tissue (*n* = 35)	Adjacent healthy endometrial tissue	RT-PCR
Hutt S et al. [51]	2019	Acta Oncologica	Literature Review	miR-944,-301	miR-205, -106b	Endometrial cancer tissue		
Li B et al. [52]	2019	J Cell Physio	Original Article		miR-148b	Endometrial cancer tissue and cell lines	Adjacent healthy endometrial tissue	RT-PCR
Li Z et al. [53]	2019	Molecular and Cellular Biochemistry	Original Article		miR-142-3p	Endometrial cancer tissue (*n* = 69)	Adjacent healthy endometrial tissue (*n* = 20)	RT-PCR
Wu et al. [54]	2019	Exp cell Res	Original Article		miR-449a	Endometrioid cancer tissue (*n* = 87)	Different cell lines	RT-PCR/immunohistochimie
Du et al. [55]	2019	int J Immunopathol Pharmacol	Original Article	miR-103		Endometrial cancer tissue (*n* = 14)		RT-PCR
Zhu et al. [56]	2019	cell Cycle	Original Article		miR-20b-5p	Endometrial cancer tissue (*n* = 36)	Adjacent healthy endometrial tissue	RT-PCR
Penolazzi L et al. [57]	2019	Gene	Original Article	miR-221		Endometrioid cancer tissue (*n* = 53), 2 groups: obese patient BMI >30kg/m^2^ and non obese patient BMI <30kg/m^2^		RT-PCR/immunohistochimie
Hu et al. [58]	2019	J cancer	Original Article		miR-449a	Endometrial cancer tissue (*n* = 40)	Endometrial cancer stage I-II	RT-PCR
liu et al. [59]	2019	oncol Lett	Original Article	hsa-miR-200b, -205, -200a, -141, -200c	hsa-miR-503,-876-3p, -144, -133a, -154	Endometrial cancer tissue (*n* = 552)	Normal endometrial tissue (*n* = 23)	RT-PCR/bioinformatics
Guo S et al. [60]	2019	Pathol Res Pract	Original Article		miR-204-5p	Endometrial cancer tissue (*n* = 22)	Adjacent healthy endometrial tissue	RT-PCR/bioinformatics (TCGA)
Liu L et al. [61]	2019	Acta Biochim Biophys Sin	Original Article		miR-27b-3p	Endometrial cancer tissue (*n* = 66)		RT-PCR/immunohistochimie
Xu et al. [62]	2019	Gynecol Oncol	Original Article		miR-30c	"Endometrial cancer tissue (*n* = 161): 141 endometrioid adenocarcinoma, 16 serous adenocarcinoma, and 4 clear cell	Adjacent healthy endometrial tissue	RT-PCR/immunohistochimie
Wang et al. [63]	2019	J Cell Biochem	Original Article	hsa-miR-1269a, -205-5p, -4652-5p, -183-3p, -183-5p, -96-5p, -182-5p, -449b-5p, -4724-5p, -891a-5p		Endometrial cancer tissue (*n* = 91)	Adjacent healthy endometrial tissue	bioinformatics TCGA
Kong et al. [64]	2019	J Int Med Res	Original Article		miR-29b	Endometrial cancer Cell lines		RT-PCR
Kozak et al. [65]	2019	Mol Cell Biochem	Original Article	miR-200 family,-141		Endometrial cancer Cell lines		RT-PCR
Dong et al. [66]	2019	J Exp Clin Cancer Res	Original Article		miR-361	Endometrial cancer Cell lines		RT-PCR
Chen P et al. [67]	2019	Biosci Rep	Original Article		miR-202	Endometrial cancer tissue (*n* = 76)	Adjacent healthy endometrial tissue	RT-PCR
Yi Su et al. [68]	2019	DNA Cell Biol.	Original Article		miR-142	Endometrial cancer tissue (*n* = 49)		RT-PCR
Hermyt et al. [69]	2019	Int J Mol Sci	Original Article	miR-331-3p, -182,-200c,-155,-200b,-874,-10a,-625,-let-7f,-let-7g,-let-7a-331,331-3p,-15b	miR-370, -432, -1296, -483-5p	Endometrial cancer tissue (*n* = 40)	Normal endometrial tissue (*n* = 20)	RT-PCR
Zheng et al. [70]	2019	Cancer Biother Radiopharm	Original Article		miR-126	Endometrial cancer tissue (*n* = 35): 28 endometrioid carcinoma, 3 serous carcinoma, 3 clear cell carcinoma, and 1 undifferentiated carcinoma	Normal endometrial tissue (*n* = 35)	immunofluorescence assay
X-C Li et al. [71]	2019	Eur Rev Med Pharmacol Sci	Original Article		miR-218	endometrial cancer tissue (*n* = 25)	Adjacent healthy endometrial tissue	RT-PCR
Asanoma et al. [72]	2019	Oncotarget	Original Article	miR-300 family		Endometrial cancer tissue (*n* = 61); 29 stage IA, 15 stage IB, 1 stage IIA, 1 stage IIB, 2 stage IIIB, 11 stage IIIC, 2 stage IVB; 56 endometrioid cancer, 5 serous carcinoma	Stage 1A endometrioid endometrial cancer (*n* = 29)	RT-PCR
Fang et al. [73]	2019	Onco target	Original Article		miR-214-3p	endometrial cancer tissue (*n* = 22)	Adjacent healthy endometrial tissue	RT-PCR
Wang et al. [74]	2019	pharmazie	Original Article		miR-363	Endometrial cancer tissue (*n* = 36)	Normal endometrial tissue (*n* = 36)	RT-PCR
Deng et al. [75]	2019	Mol Med Rep	Original Article		miR-195	Endometrial cancer Cell lines		RT-PCR
Gao et al. [76]	2019	Int J Nanomedicine	Original Article		miR-326	Endometrioid endometrial cancer tissue (*n* = 6)		RT-PCR/bioinformatics
Wang et al. [77]	2019	Cancer Biother Radiopharm	Original Article		miR-589-5p	Endometrial cancer tissue (*n* = 40)	Adjacent healthy endometrial tissue	RT-PCR
Wu et al. [78]	2019	Life Sci	Original Article	miR-616		Endometrial cancer tissue (*n* = 120)	Adjacent healthy endometrial tissue	RT-PCR
Zhang et al. [79]	2019	Kaohsiung J Med Sci	Original Article	miR-522		Endometrial cancer Cell lines		RT-PCR/bioinformatics (TCGA)
Yuan et al. [80]	2019	Reprod Sci	Original Article		miR-143,-145	Endometrial cancer Cell lines		RT-PCR
Chen et al. [81]	2019	Int J Clin Exp Pathol	Original Article	miR-135a		Endometrial cancer Cell lines		RT-PCR
Tan et al. [82]	2019	Pathol Res Pract	Original Article		miR-495	Endometrial cancer tissue (*n* = 30)		RT-PCR
Zhou et al. [83]	2019	Biosci Rep	Original Article	miR-940		Endometrial cancer tissue (*n* = 546)	Normal endometrial tissue (*n* = 33)	RT-PCR/bioinformatics (TCGA)
Li et al. [84]	2019	Gene	Original Article		miR-29c-3p	Endometrial cancer tissue (*n* = 80)	Adjacent healthy endometrial tissue	RT-PCR
Zhuang et al. [85]	2019	Endocr J	Original Article	miR-181c		Endometrial cancer Cell lines		RT-PCR
Sun et al. [86]	2019	Mol Cell Biochem	Original Article		miR-214	Endometrial cancer tissue (*n* = 27)	Normal endometrial tissue (*n* = 18)	RT-PCR
Shen et al. [87]	2019	Cell Cycle	Original Article		miR-197	Endometrial cancer tissue (*n* = 36)		RT-PCR
Zhao et al. [88]	2019	J Biosci	Original Article		miR-195	Endometrial cancer tissue (*n* = 30)	Normal endometrial tissue (*n* = 26)	RT-PCR
Liu, Y. et al. [89]	2018	Mol. Cell. Biochem	Original Article	miR-181c-3p, -25-5p	miR-99a-3p, -96a-5p, -328-3p, -337-3p, let-7c-5p	Endometrioid endometrial cancer tissue (*n* = 15)	Adjacent healthy endometrial tissue (*n* =15)	RT-PCR
Liu, Y. et al. [90]	2018	Cancer cell Int	Original Article		miR-139-5p	Endometrial cancer tissue (*n* = 25)	Normal endometrial tissue (*n* = 15)	RT-PCR
Ma, J. et al. [91]	2018	Exp. Clin. Cancer Res	Original Article		miR-302a-5p, -367-3p	Endometrial cancer tissue (*n* = 80)	Normal endometrial tissue (*n* = 80)	RT-PCR
Huang et al. [92]	2018	Biosci. Rep	Original Article	miR-106b		Endometrial cancer tissue (*n* = 20)	Normal endometrial tissue (*n* = 20)	RT-PCR
Fang et al. [93]	2018	Cell Biochem. Funct	Original Article	miR-182,-183,-153,-27a,-96		Endometrial cancer tissue (*n* = 69); 33N+, 36N−	Normal endometrial tissue (*n* = 10)	RT-PCR
Ushakov et al. [94]	2018	Bull. Exp. Boil. Med	Original Article		miR-29c,-31,-185,-652	Endometrioid endometrial cancers tissue FIGO I-II (*n* = 32)	Adjacent healthy endometrial tissue (*n* =30)	RT-PCR
Xiamei Sun [95]	2018	Mol cancer Res	Original Article	miR-652		Endometrial serous carcinoma (*n* = 13) endometrioid carcinoma (*n* = 39)	Normal endometrial tissue (*n* = 22)	RT-PCR
Yun-Xiao Zhou [96]	2018	Int J Biochem Cell Biol	Original Article	miR-146a		Endometrial cancer tissue	Normal endometrial tissue	RT-PCR
Jie Li [97]	2018	Exp ther Med	Original Article	miR-423		Endometrial cancer Cell lines		RT-PCR
Yan Li [98]	2018	J Cell Biochem	Original Article	miR-373		Endometrial cancer tissue (*n* = 64)	Adjacent healthy endometrial tissue	RT-PCR
Ying Liu [89]	2018	Mol Cell Biochem	Original Article		miR-101	Endometrial cancer tissue (*n* = 30)	Adjacent healthy endometrial tissue	RT-PCR
Xiong H [99]	2018	J Cell Biochem	Original Article	miR-183		Endometrial cancer tissue (*n* = 208)	Adjacent healthy endometrial tissue	RT-PCR
Hua Yan [100]	2018	Int J Mol Med	Original Article		miR-185-5p	Endometrial cancer tissue (*n* = 156)	Adjacent healthy endometrial tissue	RT-PCR
Yu Huang [101]	2018	Int J Clin Exp Pathol	Original Article		miR-20a-5p	Endometrial cancer tissue (*n* = 41)	Adjacent healthy endometrial tissue	RT-PCR
Ruichao Chen [102]	2018	Cell Death Dis	Original Article	miR-200c		Endometrial cancer tissue (*n* = 40)	adjacent healthy endometrial tissue	RT-PCR
Wei Bao [103]	2018	Oncol Rep	Original Article	miR-107-5p		Endometrial cancer with lymphadenectomy positive	Endometrial cancer with lymphadenectomy negative	RT-PCR
Li Yang [104]	2018	Int J Clin Exp Pathol	Original Article	miR-210		Endometrial cancer tissue (*n* = 66);49 stage FIGO I, 7 stage II, 10 stage III		RT-PCR
Yan et al. [105]	2018	Molecular Medicine Reports	Original Article	miR-944		Endometrial cancer tissue (*n* = 68); 54 endometrioid, 14 others, 55 FIGO I, 13 FIGO III-IV, 59 *n*+, 9 *n*−	Normal endometrial tissue (*n* = 20)	RT-PCR
Xie et al. [106]	2017	Cancer Biomark	Original Article		miR-216b	Endometrioid endometrial cancers tissue (*n* = 30); 12 FIGO I, 7 FIGO II, 11 FIGO III	Adjacent healthy endometrial tissue (*n* =30)	RT-PCR
Zhang et al. [107]	2017	Cancer Biomark	Original Article		miR-101	Endometrial cancer tissue (*n* = 37); 21 FIGO I, 5 FIGO II, 4 FIGO III, 5 FIGO IV	Normal endometrial tissue (*n* = 22)	RT-PCR
Chen et al. [108]	2017	Oncol. Rep	Original Article	miR-5785,-6749-5p,-1202	miR-338-3p,-449a,-196a	Endometrial cancer tissue (*n* = 15)	Normal endometrial tissue (*n* = 15)	RT-PCR
Wang Z et al. [109]	2017	Oncotarget	Original Article	miR-522, miR-139-3p, miR-520c-5p, miR-518d-5p, miR-146b-5p, miR-34a, miR-526a, miR-193a-3p, miR-221, miR-4674	miR-760	Endometrial cancer tissue	Normal endometrial tissue (*n* = 15)	RT-PCR
Liu et al. [110]	2017	Cancer biomark	Original Article	miR-1224,-1269,-182,-183, -200b, -205, -219-2, -449a, -891a, -96	miR-1-2,-100,-101-2,-1247,-133a,-139,-143,-145,-3926-1,-99a	Endometrial cancer tissue (*n* = 381)		Bioinformatics (TCGA)
Ihira et al. [111]	2017	Oncotarget	Original Article		miR-361	Endometrial cancer cell lines (*n* = 3), endometrial cancer tissue (*n* = 24)	RT-PCR	Ihira et al.
Cai et al. [112]	2016	Mol. Med. Rep	Original Article	miR-337		Endometrial cancer tissue (*n* = 24)	Adjacent healthy endometrial tissue (*n* =24)	RT-PCR
Zhao et al. [113]	2016	Oncol. Lett	Original Article		miR-126	Endometrial cancer tissue (*n* = 11)	Adjacent healthy endometrial tissue (*n* =11)	RT-PCR
Yoneyama et al. [114]	2015	Anticancer. Res	Original Article	miR-200a,-200b,-429		Endometrioid endometrial cancers tissue (*n* = 7)	Adjacent healthy endometrial tissue (*n* =7)	RT-PCR
Zhou et al. [115]	2015	Drug Des. Dev. Ther	Original Article	miR-181a		Endometrioid endometrial cancers tissue (*n* = 47); 38 FIGO I-II, 9 FIGO III-IV, 5N+, 42N−	Normal endometrial tissue (*n* = 13)	RT-PCR
Kong et al. [116]	2014	PLoS ONE	Original Article		miR-30c	Endometrioid endometrial cancers tissue (*n* = 21)	Normal endometrial tissue (*n* = 14)	RT-PCR
Jurcevic et al. [117]	2014	BMC Cancer	Original Article	miR-183, -182, 429, -135a, -9-3p, -9, 135b, -200a-5p, -218, -18a-3p	miR-1247, -199b-5p, -214, -370, -424-3p, -376c, -542-5p, -758, -377, 337-5p	Endometrial cancer tissue (*n* = 30); 10 FIGO I, 10 FIGO II, 10 FIGO III	Normal endometrial tissue (*n* = 20)	RT-PCR
Tsukamoto et al. [118]	2014	Gynecol. Oncol.	Original Article	miR-499, -135b, -205	miR-10b, -195, -30a-5p, -30a-3p, -21	Endometrioid endometrial cancers tissue (*n* = 28); 7 FIGO IA, 4N+, 21 *n*−	Normal endometrial tissue (*n* = 14)	RT-PCR
Xiong et al. [119]	2014	PLoS ONE	Original Article	miR-181c-3p, -25-5p	miR-99a-3p, -96a-5p, -328-3p, -337-3p, let-7c-5p	Endometrioid endometrial cancer tissue (*n* = 15)	Adjacent healthy endometrial tissue (*n* =15)	RT-PCR
Xu et al. [120]	2013	FEBS J.	Original Article		miR-503	Endometrial cancer tissue (*n* = 71)	Normal endometrial tissue (*n* = 5), adjacent healthy endometrial tissue (*n* = 10)	RT-PCR
Torres et al. [17]	2013	Int. J. Cancer	Original Article	miR-9, -141, -183, -200a, -200a*, -200b, -200b*, -200c, -203, -205, -429, -96, -182, -135b	miR-410	Endometrioid endometrial cancer tissue (*n* = 77): 50 FIGO I,5 FIGO II,20 FIGO III,2 FIGO IV, 29 *n*+, 15 *n*−	Normal endometrial tissue (*n* = 31)	RT-PCR
Torres et al. [121]	2012	BMC Cancer	Original Article		miR-99a, -100, -199b	Endometrioid endometrial cancer tissue (*n* = 77): 50 FIGO I,5 FIGO II,20 FIGO III,2 FIGO, 29 *n*+, 15 *n*−	Normal endometrial tissue (*n* = 31)	RT-PCR
Lee et al. [122]	2012	Mod. Pathol.	Original Article	miR-182, -183, -200a, -200c, -205		Endometrial cancer tissue (*n* = 22): 15 FIGO IA, 5 FIGO IB, 2 FIGO IIIC1	Normal endometrial tissue (*n* = 10)	RT-PCR
Karaayvaz et al. [123]	2012	PLoS ONE	Original Article	miR-200c, -205		Endometrial cancer tissue (*n* = 48); 24 endometrioid, 13 serous, 5 clear cell, 6 others	Adjacent healthy endometrial tissue (*n* =48)	RT-PCR
Zhao et al. [124]	2012	Plos One	Original Article	miR-106b-93-25		Endometrial cancer Cell lines		RT-PCR
Snowdown et al. [125]	2011	PLoS ONE	Original Article	miR-9/-9*, -18a, -96, -141, -146a, -200a/b/b*/c, -203, -205, -210, -421, -429, -516a-5p, -605, -614, -936	miR-10b*, -23a*, -100, -127-3p, -152, -199b-3p, -199b-5p, -370, 376a/c, -381, -410, -424, -424*, -431, -432, -503, -542-3/5p, -596,-610, -630, -632, -760	Endometrioid endometrial cancer tissue (*n* = 19); 9 FIGO IA, 4 FIGO IB, 1 FIGO II	Normal endometrial tissue (*n* = 10)	RT-PCR
Cohn et al. [126]	2010	Am. J. Obstet. Gynecol	Original Article	miR-9, -19b; -146, -181c, -183, -200c, -205, -223, -423, -425	let-7a, miR-32, -33b, -369, -409, -424, -431, -451, -496, -503, -516	Endometrial cancer tissue (*n* = 141); 121 endometrioid FIGO I, 3 endometrioid FIGO III, 7 serous FIGO III, 4 endometrioid FIGO IV, 6 serous FIGO IV	Normal endometrial tissue (*n* = 20)	RT-PCR
Ratner et al. [127]	2010	Gynecol. Oncol	Original Article	miR-182, -183, -200a, -205, -34a, -572, -622, -650	miR-411, -487b	Endometrioid endometrial cancer tissue (*n* = 30); 27 serous, 6 carcinosarcoma, 27 FIGO I, 12 FIGO II, 18 FIGO III	Normal endometrial tissue (*n* = 5)	RT-PCR
Chung et al. [128]	2009	Int. J. Cancer	Original Article	miR-10a, -17-5p, -23a*, -25, -28, -34a, -95, -103, -106a, -107, -130b, -141, -151, -155, -182, -183, -184, -191, -194, -200a/c, -203, -205, -210, -215, -223, -301, -325, -326, -330		Endometrioid endometrial cancer tissue (*n* = 30); 25 FIGO I–II, 5 FIGO III, 3 *n*+, 27 *n*−	Normal endometrial tissue (*n* = 22)	RT-PCR
Wu et al. [129]	2009	Eur. J. Cancer Prev	Original Article	miR-200c, -449, -205, -182, -429, -200b, -96, -31, -141, -200a, -363, -210, -432, -203, -10a, -155, -142-5p	miR-204, -193a, -368, -133b, -193b, -99b	Endometrioid endometrial cancer tissue (*n* = 10); 5 FIGO I, 5 FIGO II	Adjacent healthy endometrial tissue (*n* =10)	RT-PCR
Boren et al. [130]	2008	Gynecol. Onco	Original Article	let-7c, miR-103,-106a,-107,-181a,-185,-210,-423	let-7i, miR-30c,-152,-193,-221	Endometrioid endometrial cancer tissue (*n* = 37)	Normal endometrial tissue (*n* = 20)	RT-PCR

FIGO: International Federation of Gynecology and Obstetrics, *n*: ganglionic status, RT-PCR: real-time polymerase chain reaction.

**Table 2:** DNA methylation levels of miRNA loci in endometrial tissues.

Reference	Date	Type of Review Article	Tumor Suppressor or OncomiR	miRNA	DNA Methylation Level	Methylation Target	Case Sample		Case Control	Detection Technique
Yang et al. [14]	2020	Original article	OncomiR	miR-191	hypomethylation	APC via TET1	Endometrial Cell lines			Hydroxymethylated DNA Immunoprecipitation quantitative PCR
Ni J et al. [22]	2020	Original article	tumor supressor	miR-638	hypermethylation	miRNA loci	Endometrial cancer tissue (*n* = 68)	Normal endometrial tissue (*n* = 68)		bisulfite sequencing/PCR
Zhang et al. [23]	2018	Original article	Tumor supressor	miR-137	hypermethylation	miRNA loci	Endometrial cancer tissue (*n* = 67)	Normal endometrial tissue (*n* = 10)		COBRA assay
Yanokura et al. [24]	2017	Original article	Tumor supressor	miR-663	hypermethylation	miRNA loci	Endometrial cancer tissue (*n* = 25)	Adjacent healthy tissue (*n* = 25)		bisulfite sequencing/PCR
Devor et al. [25]	2015	Original article	OncomiR	miR-182	hypomethylation	miRNA loci	Endometrial cancer tissue (*n* = 34); 18 endometrioid, 16 serous	Normal endometrial tissue (*n* = 6)		bisulfite sequencing/PCR
Moreno-Moya et al. [139]	2014	Original article	OncomiR	miR-30d	hypermethylation	H19	Endometrial epithelial cell (*n* = 4)			anti-5-methylcytosine monoclonal antibody
Li et al. [135]	2013	Original article	OncomiR	miR-200b	hypomethylation	miRNA loci	lynch endometrial cancer tissue (*n* = 19), other endometrial cancer tissue (*n* = 64)	Normal endometrial tissue (*n* = 22)		bisulfite sequencing/PCR
Li et al. [135]	2013	Original article	OncomiR	miR-130a/b	hypomethylation	miRNA loci	lynch endometrial cancer tissue (*n* = 19), other endometrial cancer tissue (*n* = 64)	Normal endometrial tissue (*n* = 22)		bisulfite sequencing/PCR
Li et al. [135]	2013	Original article	OncomiR	miR-625	hypomethylation	miRNA loci	lynch endometrial cancer tissue (*n* = 19), other endometrial cancer tissue (*n* = 64)	Normal endometrial tissue (*n* = 22)		bisulfite sequencing/PCR
Li et al. [135]	2013	Original article	OncomiR	miR-222	hypomethylation	miRNA loci	lynch endometrial cancer tissue (*n* = 19), other endometrial cancer tissue (*n* = 64)	Normal endometrial tissue (*n* = 22)		bisulfite sequencing/PCR
Hiroki et al. [136]	2012	Original article	Tumor supressor	miR-34b	hypermethylation	miRNA loci	Endometrial cancer tissue (*n* = 41)	Normal endometrial tissue (*n* = 7)		bisulfite sequencing/PCR
Pavicic et al. [137]	2011	original article	Tumor supressor	miR-124a-1		miRNA loci	Endometrial cancer tissue (*n* = 41)	Normal endometrial tissue (*n* = 41)		bisulfite sequencing/PCR
Pavicic et al. [137]	2011	original article	Tumor supressor	miR-124a-2	hypermethylation	miRNA loci	Endometrial cancer tissue (*n* = 41)	Normal endometrial tissue (*n* = 41)		bisulfite sequencing/PCR
Pavicic et al. [137]	2011	original article	Tumor supressor	miR-124a-3	hypermethylation	miRNA loci	Endometrial cancer tissue (*n* = 41)	Normal endometrial tissue (*n* = 41)		bisulfite sequencing/PCR
Tsuruta et al. [21]	2011	original article	Tumor supressor	miR-152	hypermethylation	miRNA loci	Endometrial cancer tissue (*n* = 70); 38 stage I, 8 stage II, 21 stage III, 3 stage IV			bisulfite sequencing/PCR
Pavicic et al. [137]	2011	Original article	OncomiR	miR-208a	hypomethylation	miRNA loci	Endometrial cancer tissue (*n* = 41)	Normal endometrial tissue (*n* = 41)		bisulfite sequencing/PCR
Huang et al. [138]	2009	original article	Tumor supressor	miR-129-2	hypermethylation	miRNA loci	Endometrial cancer tissue (*n* = 117)	Normal endometrial tissue (*n* = 7)		bisulfite sequencing/PCR

COBRA: The Combined Bisulfite Restriction Analysis; PCR: polymerase chain reaction.

## Data Availability

No new data were created or analyzed in this study. Data sharing is not applicable to this article.

## Appendix A

**Table A1:** MicroRNAs Regulation of epigenetic modifiers in endometrial cancer.

miR	Increased Expression level in Endometrial Cancer	Decreased Expression Level in Endometrial Cancer	Methylation Modified	Reference
miR-1		X		[169]
miR-1-1		X		[137]
miR-1-2		X		[110]
miR-6		X		[168]
miR-7	X			[169]
let-7a	X			[29]
let-7c		X		[29]
let-7f	X			[29]
let-7g	X			[29]
miR-9	X			[117,121,125,126]
miR-9*	X			[125]
miR-9-3p	X			[117]
miR-10a	X			[128,129,169]
miR-10b*		X		[125,169]
miR-10b	X	X		[118]
miR-15a		X		[47,170]
miR-15b	X			[25,29]
miR-17	X			[136]
miR-17-5p	X			[128]
miR-18a	X			[125,136]
miR-18a-3p	X			[117]
miR-18b	X	X		[137,169]
miR-19b	X			[126]
miR-20a-5p		X		[101]
miR-20b-5p		X		[56]
miR-21		X		[118]
miR-21-5p	X			[27]
miR-23a	X			[128]
miR-23a*	X	X		[125,169]
miR-23b		X		[169]
miR-23b-5p		X		[17]
miR-23c		X		[17]
miR-24-1*		X		[169]
miR-25	X			[128]
miR-25p	X			[142]
miR-26a	X			[126]
miR-26a1	X			[126]
miR-27a	X			[171,172]
miR-27b		X		[136]
miR-27b-3p		X		[89]
miR-28	X			[128]
miR-29b		X		[169]
miR-29c		X		[50]
miR-29c-3p		X		[48]
miR-30a-5p		X		[118]
miR-30a-3p		X		[134]
miR-30c		X		[116,130,173]
miR-30c2*	X			[169]
miR-30d	X		X	[139]
miR-31	X	X		[129,169]
miR-32		X		[126]
miR-33a		X		[169]
miR-33b		X		[128]
miR-33b*	X			[169]
miR-34a	X	X		[109,127,128]
miR-34b		X	X	[136]
miR-34b*		X		[169]
miR-34c		X		[140,174]
miR-34c-3p	X	X		[169]
miR-34c-5p		X		[169]
miR-95	X			[128,169]
miR-96	X			[121,125,129,169]
miR-98	X			[12]
miR-99a		X		[121,143]
miR-99a-3p		X		[119]
miR-99b		X		[129]
miR-100		X		[121,125]
miR-101		X		[169]
miR-101-2		X		[110]
miR-103	X			[128,130]
miR-106a	X			[29,128,130,136]
miR-106b		X		[92,175]
miR-106b-93-25	X			[124]
miR-107	X			[128,130,176]
miR-107-5p	X			[103]
miR-123	X			[126]
miR-124a-1		X	X	[137]
miR-124a-2		X	X	[137]
miR-124a-3		X		[137]
miR-125a-3p	X			[169]
miR-125b-1	X			[126]
miR-125b-2	X	X		[177]
miR-126		X		[113]
miR-127-3p		X		[125,169]
miR-129-2		X	X	[138]
miR-129-3p		X		[25]
miR-129-5p		X		[169]
miR-130a			X	[135]
miR-130b	X	X	X	[128]
miR-132*		X		[169]
miR-133a	X	X		[25,169]
miR-133b		X		[129,169,177]
miR-134	X			[169]
miR-135a*	X			[29,169]
miR-135a	X			[117,177]
miR-135b	X			[29,117,118,121,169,177,178]
miR-136		X		[169]
miR-136*		X		[169]
miR-137		X	X	[23,25]
miR-139		X		[110]
miR-139-3p	X			[161]
miR-139-5p		X		[90,169]
miR-140-3p		X		[169]
miR-140-5p		X		[169]
miR-141	X			[29,121,125,128,178]
miR-142		X		[68]
miR-142-3p	X	X		[136]
miR-142-5p	X	X		[129,177]
miR-143	X	X		[161,169]
miR-143*		X		[169]
miR-145		X		[161,169]
miR-145*		X		[169]
miR-145a	X			[126]
miR-146	X			[126]
miR-146a	X	X		[126]
miR-148a		X		[137,173]
miR-148a-3p	X			[169]
miR-148b		X		[52]
miR-150*	X			[136]
miR-151	X			[128]
miR-152		X	X	[21,125,130,137,169]
miR-153	X			[100]
miR-155	X			[128,129,175]
miR-181a	X			[12,115,130]
miR-181c	X			[126]
miR-181c-3p	X			[119]
miR-182	X		X	[25,29,121,122,127,128,129,169,178]
miR-183	X			[29,122,126,127,128,169,178]
miR-183-3p	X			[13]
miR-183-5p	X			[13]
miR-184	X	X		[128,162]
miR-185	X	X		[94,130]
miR-185a	X			[168]
miR-185-5p	X			[13]
miR-186	X			[29,47]
miR-188-5p	X			[169]
miR-190b	X			[132]
miR-191	X		X	[14,128]
miR-193		X		[130]
miR-193a		X		[129]
miR-193a-3p	X			[109]
miR-193a-5p	X			[169]
miR-193b		X		[129]
miR-194	X	X		[128,179,180]
miR-195		X		[118,169]
miR-196a-5p		X		[119]
miR-196b		X		[169]
miR-197		X		[87]
miR-198	X			[169]
miR-199a-5p		X		[169]
miR-199b-5p		X		[117,125,169]
miR-199b-3p		X		[125,169]
miR-199b		X		[121]
miR-200		X		[181]
miR-200a	X			[29,114,121,125,127,128,129,169,177,178]
miR-200a-5p	X			[117]
miR-200a*	X			[121,169,178]
miR-200b	X		X	[29,114,121,125,129,169,174,177]
miR-200b*	X			[125,169,178]
miR-200c	X	X		[29,40,121,125,126,128,129,169,177,178]
miR-202	X	X		[141,169]
miR-202-3p		X		[67]
miR-203	X			[121,125,128,129,169,177]
miR-203		X		[142]
miR-204	X	X		[10,54,182]
miR-204-5p		X		[103]
miR-205	X			[29,118,121,123,125,126,127,128,129,169,177,178]
miR-205-5p		X		[36]
miR-208a			X	[137]
miR-210	X			[125,128,129,130,169,177]
miR-214		X		[117,169]
miR-214*		X		[169]
miR-214-3p		X		[25]
miR-215	X			[128]
miR-216b		X		[178]
miR-218	X			[117]
miR-219-2	X			[110]
miR-221	X			[130]
miR-222	X		X	[29,140,182]
miR-222-3p	X			[170]
miR-223	X			[29,126,128,140,169]
miR-224	X			[169]
miR-299-3p		X		[169]
miR-299-5p		X		[169]
miR-301	X			[51,128]
miR-302a-5p		X		[91]
miR-320a		X		[33]
miR-325	X			[128]
miR-326	X	X		[110,128]
miR-328-3p		X		[119]
miR-330	X			[128]
miR-330-3p	X			[169]
miR-331	X			[54]
miR-331-3p	X			[69]
miR-335	X			[39]
miR-337-3p		X		[119]
miR-337-5p		X		[117,169]
miR-340-5p	X			[33]
miR-361		X		[66]
miR-363	X			[129]
miR-368		X		[129]
miR-369		X		[126]
miR-370		X		[117]
miR-371-5p	X			[169]
miR-373	X			[97]
miR-375	X			[169]
miR-376a		X		[125,169]
miR-376c		X		[117,125,169]
miR-377		X		[117,169]
miR-379		X		[169]
miR-381		X		[125,169]
miR-382		X		[146]
miR-409		X		[126]
miR-410		X		[121,125,169]
miR-411		X		[127,169]
miR-421	X			[125]
miR-423	X			[126,130]
miR-424		X		[125,126,169]
miR-424*		X		[125]
miR-424-3p		X		[117]
miR-425	X			[126,169]
miR-429	X			[114,117,121,125,129,169,177]
miR-431		X		[125,126]
miR-432	X	X		[125,129]
miR-449	X			[29,129]
miR-450a		X		[169]
miR-451		X		[126]
miR-455-3p		X		[169]
miR-455-5p		X		[169]
miR-483-5p	X			[169]
miR-487b		X		[127]
miR-494	X			[169]
miR-495		X		[132]
miR-496		X		[126]
miR-497		X		[169]
miR-499	X			[118]
miR-501-5p	X			[169]
miR-503		X		[120,125,126,169]
miR-504		X		[169]
miR-505*	X			[169]
miR-513a-5p	X			[169]
miR-513b	X			[169]
miR-516		X		[126]
miR-516a-5p	X			[125]
miR-518c*	X			[169]
miR-519a	X			[177]
miR-520-5p	X			[161]
miR-522	X			[161]
miR-526a	X			[161]
miR-542-3p		X		[125,169]
miR-542-5p		X		[117,125,169]
miR-543		X		[183]
miR-557	X			[169]
miR-564	X			[169]
miR-572	X			[127]
miR-575	X			[169]
miR-589-5p		X		[13]
miR-596		X		[125]
miR-601	X			[136]
miR-605	X			[125]
miR-610		X		[125]
miR-614	X			[125]
miR-616	X			[54]
miR-622	X			[127,169]
miR-623	X			[169]
miR-625	X		X	[54,135]
miR-629*	X			[169]
miR-630	X	X		[125,169]
miR-632		X		[125]
miR-638		X	X	[22]
miR-646		X		[31]
miR-650	X			[127]
miR-652	X			[169]
miR-654-3p		X		[169]
miR-663	X		X	[24,169]
miR-758		X		[117]
miR-760	X	X		[125,169]
miR-765	X			[169]
miR-768-5p	X			[169]
miR-801	X			[169]
miR-873		X		[169]
miR-874	X			[95]
miR-876		X		[59]
miR-877	X			[169]
miR-892b	X			[169]
miR-888	X			[177]
miR-891a	X			[110]
miR-923	X			[169]
miR-936	X			[125]
miR-939	X			[169]
miR-940		X		[89]
miR-944	X			[51,105]
miR-1202	X			[108]
miR-1224	X			[110]
miR-1224-5p	X			[169]
miR-1225-5p	X			[169]
miR-1226	X			[169]
miR-1228	X			[121]
miR-1247		X		[117]
miR-1269	X			[13]
miR-1271		X		[184]
miR-1976		X		[140]
miR-3170		X		[140]
miR-3926-1		X		[110]
miR-4461		X		[27]
miR-4467		X		[169]
miR-4758	X			[28]
miR-5001-5p		X		[169]
miR-5787	X			[108]
miR-6076		X		[34]
miR-6511b		X		[27]
miR-6749-5p	X			[108]
miR-6950-5p		X		[169]

X = when the miRNA is increased, decreased or if the methylation of miRNA’s loci is modified.
